# The Novel Tubulin Polymerization Inhibitor MHPT Exhibits Selective Anti-Tumor Activity against Rhabdomyosarcoma *In Vitro* and *In Vivo*

**DOI:** 10.1371/journal.pone.0121806

**Published:** 2015-03-26

**Authors:** Yan Mu, Yin Liu, Liwen Li, Cong Tian, Hongyu Zhou, Qiu Zhang, Bing Yan

**Affiliations:** School of Chemistry and Chemical Engineering, Shandong University, Shandong Province, Jinan, China; Ospedale Pediatrico Bambino Gesu', ITALY

## Abstract

The dose-limiting toxicity caused by standard chemotherapy has become a major roadblock to successful rhabdomyosarcoma chemotherapy. By screening a thiazolidinone library including 372 compounds, a novel synthetic compound, 2-((4-hydroxyphenyl)imino)-5-(3-methoxybenzylidene)thiazolidin-4-one (MHPT), was identified as a potent and selective anti-rhabdomyosarcoma agent. MHPT inhibited 50% of the growth of the rhabdomyosarcoma cell lines RD and SJ-RH30 at 0.44 μM and 1.35 μM, respectively, while displaying no obvious toxicity against normal human fibroblast cells at 100 μM. Further investigation revealed that MHPT suppressed the polymerization of tubulin, leading to rhabdomyosarcoma cell growth arrest at the G2/M phase followed by apoptosis. *In vivo*, MHPT inhibited tumor growth by 48.6% relative to the vehicle control after 5 intraperitoneal injections of 40 mg/kg without appreciable toxicity to normal tissues and systems in an RD xenograft mouse model, while vincristine caused lethal toxicity when similar growth inhibition was achieved. As a moderate tubulin polymerization inhibitor compared with vincristine, MHPT requires a more dynamic tubulin to exert its cytotoxicity, which is a situation that only exists in cancer cells. This attribute may account for the low toxicity of MHPT in normal cells. Our data suggest that MHPT has the potential to be further developed into a selective anti-rhabdomyosarcoma drug with low toxicity.

## Introduction

Rhabdomyosarcoma (RMS) is the most common soft tissue sarcoma in children. It develops through disruption of muscle differentiation and may arise at any site within the human body [1–3]. RMS is divided into two major subtypes: embryonal rhabdomyosarcoma (ERMS) and alveolar rhabdomyosarcoma (ARMS). Despite improvements in multidisciplinary therapeutic approaches, the RMS survival rate is still unsatisfactory, and survivors suffer from treatment-related morbidity or mortality [4–6]. The standard chemotherapy adopted by Children's Oncology Group and the Intergroup RMS Study Group (IRSG) is the three-drug combination of vincristine (VCR), dactinomycin and cyclophosphamide [7]. However, these traditional chemotherapy drugs can lead to serious toxic side effects such as weight loss, neurotoxicity, leukopenia, thrombocytopenia and hair loss, which greatly limit their clinical applications [8, 9]. These drugs can even cause death due to their severe toxicity [10]. Therefore, it is urgently necessary to develop selective and safe anti-RMS agents.

Thiazolidinone derivatives have been demonstrated to be active as antibacterial, antifungal, anti-inflammatory, anticoagulant and anti-HIV agents; however, their bioactivity against tumors has not been well investigated [11]. To search for potential anti-tumor agents with high efficacy and safety, a thiazolidinone library including 372 compounds was designed and synthesized. Several hit compounds were identified as potent anti-lung cancer agents in our previous study [12, 13]. In this study, compounds in this thiazolidinone library were evaluated for their anti-RMS activity in the human ERMS cell line RD and ARMS cell line SJ-RH30 with the aim of for developing potent and selective drugs. The cytotoxicity of the hit compounds against normal cells was also evaluated using the normal human fibroblast cell lines NHFB, MRC-5 and WI-38. One compound, 2-((4-hydroxyphenyl)imino)-5-(3-methoxybenzylidene)thiazolidin-4-one (MHPT), was identified as the most potent and highly selective anti-RMS agent. In the RD xenograft tumor model, MHPT significantly inhibited the growth of tumors without appreciable toxicity. The mechanistic study demonstrated that MHPT induced cell cycle arrest at the G2/M phase followed by apoptosis in RD and SJ-RH30 cells by depolymerizing microtubules. Our data suggested that MHPT has the potential to be further developed into a candidate anti-RMS candidate drug.

## Materials and Methods

### Reagents

The cell proliferation reagent WST-1 (#C0036), RIPA lysis buffer (#P0013C) and the BCA protein assay kit (#P0012) were purchased from Beyotime Institute Biotechnology (Songjiang, Shanghai, China). Guava Viacount (#4000–0040), Cell Cycle (#4500–0220) and Nexin reagents (#4500–0450) were purchased from Millipore Corporation (Billerica, MA, USA). Apo-ONE homogeneous caspase-3/7 assay kit (#G7792) was purchased from Promega (Madison, WI, USA). The fluorescence based tubulin polymerization assay kit (#BK011P) was purchased from Cytoskeleton (Denver, CO, USA). The anti-p21 (#2947), anti-PARP (#9542), anti-α-tubulin (#2144) and anti-β-actin (#4967) antibodies were purchased from Cell Signaling Technology (Beverly, MA, USA). The goat anti-mouse (#170–6516) or goat anti-rabbit (#170–6515) secondary antibodies were purchased from LI-COR Biotechnology (Lincoln, NE, USA). VCR and other chemical reagents were obtained from Sigma-Aldrich (St. Louis, MO, USA).

### Cell culture

The ERMS cell line RD and the normal human fibroblast cell line NHFB were purchased from the Cell Bank of the Type Culture Collection of the Chinese Academy of Sciences (Songjiang, Shanghai, China). The normal human fibroblast cell lines MRC-5 and WI-38, and the ARMS cell line SJ-RH30 were provided by St. Jude Children’s Research Hospital (Memphis, TN, USA) who purchased the three cell lines from the American Type Culture Collection (Rockville, MD, USA). The cells were cultured in DMEM supplemented with 10% fetal calf serum, 100 units/mL penicillin, and 100 mg/mL streptomycin. The media and culture reagents were purchased from Gibco (Grand Island, NY, USA). Both cell lines were maintained in a 37°C incubator with 95% humidity and 5% CO_2_. Cells used in the experiments were collected in the logarithmic phase.

### Cell proliferation assay

Cells were seeded in 96-well microtiter plates (6000 cancer cells and 8000 normal cells in 100 μL of culture medium per well). After 24 h, the cells were treated with compounds from thiazolidinone library or with an equal volume (0.1%) of DMSO as a control. After 48 h, the WST-1 working solution was added to each well and incubated for 2 h. The absorbance of each well was determined at 450 nm by using a microplate reader (Model 680, Bio-Rad, USA).

### Cell morphological changes and viable cell count

Cells were seeded in 6 cm flat-bottom culture plates (180,000 cells in 3 mL of culture medium). After 24 h, the cells were treated with 5 μM MHPT or DMSO. Morphological changes in the cells were observed, and the cells were photographed at 6, 12, 24 and 48 h by using an Olympus IX 71 phase-contrast microscope (Center Valley, PA, USA). Then, these cells were harvested and stained with Guava Viacount reagent for counting viable cells [14]. The stained cell samples were analyzed by flow cytometry (Guava EasyCyte, Millipore, USA).

### Cell cycle analysis

Cells were seeded in 6 cm flat-bottom culture plates. After 24 h, the cells were treated with MHPT at the indicated concentrations for 24 h or at 5 μM for various times. The cells were harvested and fixed in 70% ethanol at -20°C overnight. After washing with PBS, the cells were stained with Guava cell cycle reagent for cell cycle analysis and analyzed by flow cytometry [15].

### Apoptosis detection

Cells were seeded in 6 cm flat-bottom culture plates. After 24 h, the cells were treated with MHPT at the indicated concentrations for 48 h or at 5 μM for various times. Then, the cells were washed with PBS, stained with Guava Nexin reagent (containing Annexin V-PE and 7-AAD) and analyzed by flow cytometry [16].

### Caspase 3/7 activity assay

Caspase 3/7 activity was determined in RD cells based on fluorescence intensity [17]. RD cells were seeded in a 96-well microtiter plate (8000 cells in 100 μL per well). After 24 h, the cells were treated with MHPT (1 μM or 5 μM) or DMSO. After the cells had been treated for 6, 12, 24 and 48 h, the Apo-ONE homogeneous caspase 3/7 assay working solution was dispensed into each well and incubated for 1 h at room temperature. The fluorescence of each well was determined at a wavelength of 499 nm with an emission wavelength of 521 nm using a multimode plate reader (Victor X2, PerkinElmer, USA). The fluorescence intensity is proportional to the activity of caspase 3/7.

### Tubulin polymerization assay

A fluorescence based tubulin polymerization assay kit was applied to monitor the polymerization process as described by the manufacturer and the literatures [18]. Tubulin (> 99% pure) was purified from bovine brain tissue. Briefly, 5 μL of control buffer (5% DMSO in general tubulin buffer) or MHPT was added into each well of the assay plate at the indicated concentrations after pre-warming the plate at 37°C for 1 min. Then, tubulin solution (50 μL) was dispensed rapidly into each well. The polymerization dynamics of tubulin were monitored for 60 min at 37°C by measuring the change in fluorescence every 2 min using a 1420 Multi-label Counter (Perkin Elmer, USA). The excitation was 355 nm, and the emission was 460 nm.

### Western blot analysis

After treatment with MHPT or DMSO, the cells were washed and collected. For analysis of p21 and PARP proteins, cell lysates were prepared conventionally in RIPA lysis buffer [19]. For analysis of tubulin protein, polymeric (cytoskeletal) and monomeric (soluble) tubulin were prepared as previously described [20]. Tubulin was extracted under conditions that prevent microtubule depolymerization (0.1% Triton X-100; 0.1 M N-morpholinoethanesulfonic acid, pH 6.75; 1 mM MgSO_4_; 2 mM EGTA; and 4 M glycerol). After centrifugation, monomeric soluble tubulin was collected in the liquid supernatant. The remaining precipitate was dissolved in 0.5% SDS in 25 mM Tris (pH 6.8) and cytoskeletal tubulin (polymeric tubulin) was extracted. Total protein was quantified using the BCA protein assay. Equal amounts of total protein were loaded onto 10% SDS-PAGE gels and transferred onto PVDF membranes. After blocking with 5% nonfat milk in TBS/T (TBS containing 0.1% Tween 20) for 60 min, the membrane was blotted with primary antibodies against p21, PARP, α-tubulin and β-actin at 1:1000 dilutions overnight at 4°C. The membranes were then probed with goat anti-mouse or goat anti-rabbit secondary antibodies (1:5000) at room temperature for 60 min. The immunoblots were visualized by enhanced chemiluminescence.

### *In vivo* experiments

The inhibitory effect of MHPT on human ERMS RD tumors was examined in nude mice. All procedures involving animals and their care were approved by the Institutional Animal Care and Use Committee of Shandong University. The mice were sacrificed under sodium pentobarbital anesthesia, and all efforts were made to minimize suffering.

BALB/C nude mice (female, 4 weeks old) were used to construct an RD xenograft tumor model. After one week of acclimatization, RD cells (10 million in 100 μL of DMEM) were inoculated subcutaneously into the lower right dorsal flank of the mice. Tumor volume was calculated according to a standard formula:$Volume=\frac{Width^{2}\times Length}{2}$. When the tumors grew to 500 mm^3^, the xenografts were excised from the mice and cut into 1–2 mm^3^ blocks in a sterile environment. Fresh tumor blocks were inoculated into the lower right dorsal flank of BALB/C nude mice (female, 4 weeks old). When these subcultured tumors grew to 100 to 150 mm^3^, 15 mice were randomized into three groups for different treatments: Control (vehicle including 85% PBS and 15% PPG), MHPT, and VCR. Five doses of MHPT were administered intraperitoneally every other day (40 mg/kg each dose, total of 200 mg/kg), which was a safe dose based on the acute toxicity experiment (see Supporting Information). Five doses of VCR were administered intraperitoneally every 4 days (1 mg/kg each does) based on the literatures [21–24]. MHPT and VCR therapy were stopped if serious adverse reactions occurred in the treated mice (weight loss of over 20% or other obvious signs of suffering). Tumor volumes and body weights were measured every other day. When the tumors grow to nearly 2000 mm^3^, the mice were sacrificed. Tumor growth inhibition (TGI) rates were assessed by the following formula:$TGI=1-\frac{T_{t}-T_{0}}{C_{t}-C_{0}}$. *T*_*t*_ and *T*_*0*_ indicate the tumor volumes of the treated group at day t and day 0. *C*_*t*_ and *C*_*0*_ indicate the tumor volumes of the control group at day t and day 0. The blood of each mouse was utilized for biochemical and hematological analyses. Major organs (including the heart, liver, spleen, lung, and kidney) were collected and fixed in 10% formalin overnight. Paraffin-embedded tissue sections were prepared and stained with H&E using standard procedures. These tissue sections were examined under a microscope by a veterinary pathologist.

### Statistical analysis

Data are expressed as the mean ± SD. The statistical significance was tested using an unpaired two-tailed Student’s *t*-test. *P*-values less than 0.05 were considered to indicate statistically significant differences.

## Results

### MHPT has selective cytotoxicity against RMS cells

**Fig 1:**
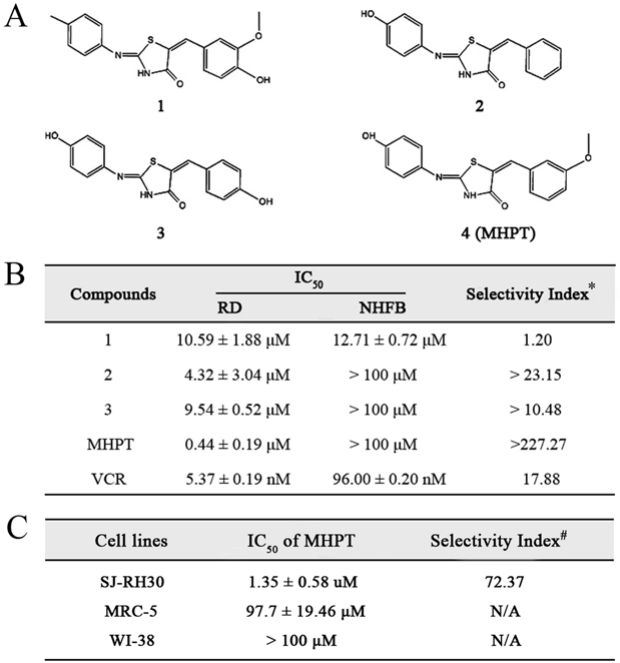
Cytotoxicity of compounds in RMS cells and normal human fibroblast cells. (A) Chemical structures of anti-RMS hits. (B) IC_50_ values of the compounds in RD and NHFB cells. *, The selectivity index was calculated by dividing the IC_50_ in NHFB cells by the IC_50_ in RD cells. (C) The IC_50_ values of MHPT in SJ-RH30 RMS cells and the normal human fibroblast cell lines MRC-5 and WI-38. #, the selectivity index was calculated by dividing the IC_50_ in MRC-5 cells by the IC_50_ in SJ-RH30 cells.

A thiazolidinone library containing 372 compounds was designed and synthesized as previously reported [12]. In this study, the anti-RMS activity of these thiazolidinone compounds was evaluated by high-throughput cytotoxicity screening with the aim of developing potent and selective drugs. Screening was carried out in two stages: primary screening and dose-response determination. In the primary screening of the library, human ERMS RD cells were exposed to the compounds at a concentration of 10 μM for 48 h, and cell viability was determined with the WST-1 cell proliferation assay (Fig. 1A). Compared with DMSO, the hits that caused over 50% viability loss were selected for the dose-response determination (Fig. 1B). Dose-limiting toxicity is one important roadblock to successful RMS chemotherapy. The cytotoxicity of four hits against normal human cells (NHFB) was determined. Among these four hits, MHPT demonstrated the greatest cytotoxicity against RD cells (IC_50_ = 0.44±0.19 μM) but had no obvious toxicity against NHFB cells (IC_50_ >100 μM). The selectivity index of compounds in Fig. 1B was calculated by dividing the IC_50_ against NHFB cells by the IC_50_ against RD cells. The selectivity index of MHPT (227) was far higher than that of the other three hits and that of the clinical drug VCR (17.88), which indicates that MHPT is much safer than these other compounds. To confirm the selective cytotoxicity of MHPT, its cytotoxicity was further evaluated in another RMS cell line, SJ-RH38, and two other normal human fibroblast cell lines, MRC-5 and WI-38. In these experiments, MHPT also showed great selective cytotoxicity against SJ-RH38 cells (Fig. 1C).

**Fig 2:**
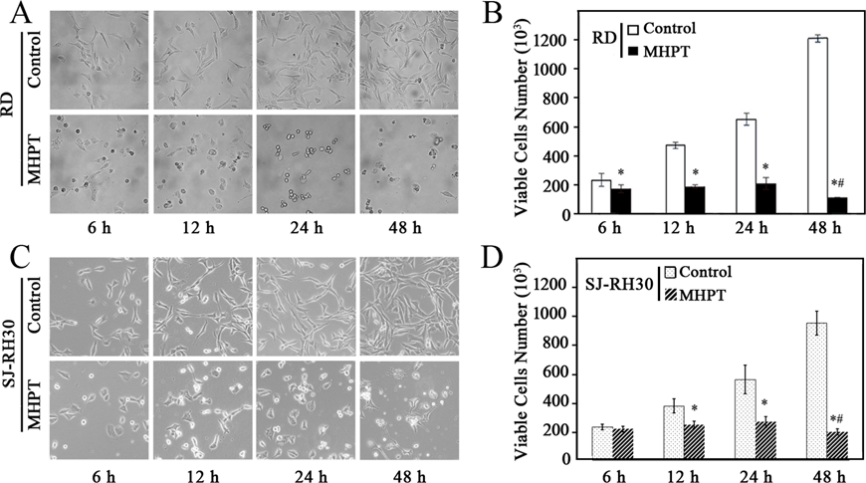
Time-dependent morphological changes (A, C) and changes in viability (B, D) of RMS cells treated with DMSO (control) or MHPT (5 μM). DMSO- treated RMS cells grew exponentially; however the growth of MHPT-treated RMS cells was inhibited significantly. The number of viable RMS cells decreased significantly after treatment with MHPT for 48 h. *, *P* < 0.05 versus control; #, *P* < 0.05 versus MHPT at 24 h.

To further investigate the cytotoxicity of MHPT, both morphological changes and viability were examined in RD and SJ-RH30 cells. When treated with 5 μM MHPT, the cells detached, became rounded, and shrank to form irregular spheres (Fig. 2A, 2C). We stained the viable cells with Guava Viacount reagent and found that MHPT halted RMS cell proliferation significantly, and some of the RMS cells died after long treatment duration (48 h). In contrast, RMS cells treated with DMSO grew rapidly and exponentially (Figs. 2B and 2D). Taken together, our data indicate that MHPT is a novel anti-RMS compound with high potency and selectivity.

### MHPT caused cell cycle arrest and apoptosis in RMS cells

**Fig 3:**
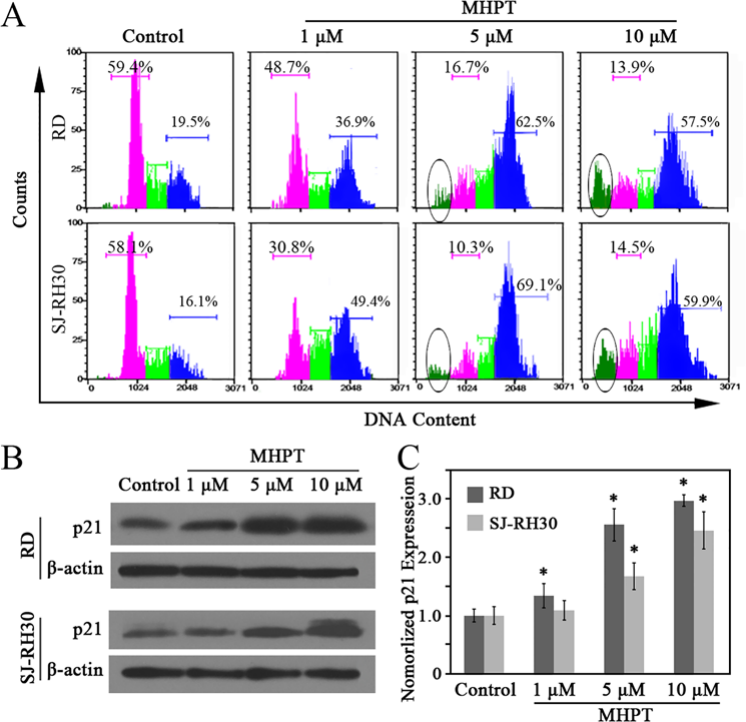
MHPT caused G2/M cell cycle arrest of RMS cells in a dose-dependent manner. RMS cells were treated with DMSO (control) or MHPT at the indicated concentrations for 48 h. (A) RMS cells were stained and analyzed by a flow cytometry. The pink areas indicate arrest at the G1/G0 phase; the green areas indicate arrest at the S phase, the blue areas indicate arrest at the G2/M phase; and the circled areas indicate arrest at the sub-G1 phase. (B) Western blot analysis of p21 expression after MHPT treatment in RMS cells. (C) The ratio of p21 to β-actin was quantified by densitometric analysis using Image J software and normalized to the value of the control group. *, *P* < 0.05 versus control.

To further investigate the anti-cancer activity of MHPT in RMS cells, the cell cycle distribution of RD and SJ-RH30 cells was examined. MHPT treatment caused a rapid, dose-dependent, and strong cell cycle arrest in the G2/M phase (Fig. 3A) in both cell lines. Approximately 60–70% of RMS cells were arrested at G2/M phase in the presence of a high concentration of MHPT. G2/M arrest induced by MHPT in RD cells was also time-dependent (S1 Fig.). Protein p21, an inhibitor of cell cycle progression [25], was analyzed by Western blot. After MHPT treatment, p21 was significantly up-regulated in a dose-dependent manner in RMS cells (Figs. 3B and 3C).

**Fig 4:**
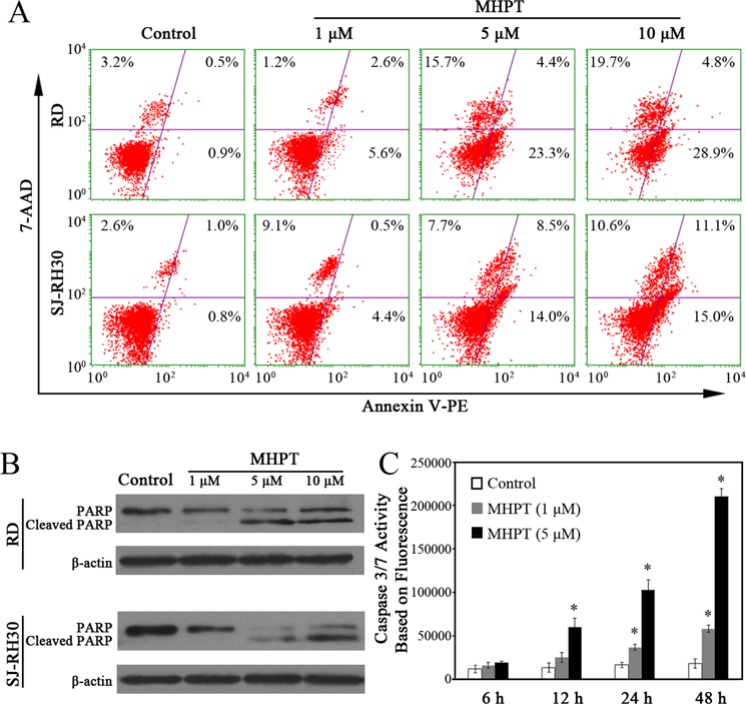
MHPT induced RMS cell apoptosis in a dose-dependent manner. (A) RMS cells were treated with DMSO (control) or MHPT at the indicated concentrations for 48 h. The RMS cells were stained by Guava Nexin regent containing 7-AAD/Annexin-V-PE and analyzed by flow cytometry. In the four windows of each plot, the lower left indicates normal cells, the lower right indicates early apoptotic cells, and the upper right indicates late-phase apoptotic cells or necrotic cells. (B) Western blot analysis of cleaved PARP after MHPT treatment in RMS cells. (C) Caspase 3/7 activity was determined based fluorescence intensity in RD cells. RD cells were treated with DMSO (control) or MHPT (1 μM or 5 μM) for the indicated times. MHPT significantly activated caspase 3/7 in a dose- and time-dependent manner. *, *P* < 0.05 versus control.

A sub-G1 peak is generally thought to be induced in populations of apoptotic cells. Here, a noticeable increase in cells in the sub-G1 phase was detected after 24 h of treatment (Fig. 3A). To further validate the occurrence of apoptosis, the proportion of apoptotic cells was evaluated by flow cytometry, the cleavage of PARP was examined by Western blot, and a caspase 3/7 activity assay was performed. The flow cytometry data showed that, after 48 h of treatment, MHPT induced apoptosis in RD and SJ-RH30 cells in a dose-dependent manner. Approximately 28% of the RD cells and 23% of the SJ-RH30 cells were apoptotic/necrotic in the presence of 5 μM MHPT (Fig. 4A). The induction of apoptosis by MHPT in RD cells was also time-dependent (S2 Fig.). Caspase 3 and caspase 7 are the critical mediators of apoptosis. We validated the occurrence of apoptosis by examining the cleavage of PARP (a well-known caspase 3 substrate) by Western blot (Fig. 4B) and by examining caspase 3/7 activation using fluorescence experiment (Fig. 4C). The data showed that caspase 3/7 was significantly activated in a dose- and time-dependent manner.

Taken together, our data indicate that MHPT caused G2/M cell cycle arrest followed by apoptosis in RD and SJ-RH30 cells in a dose- and time-dependent manner.

### Tubulin polymerization was inhibited by MHPT

**Fig 5:**
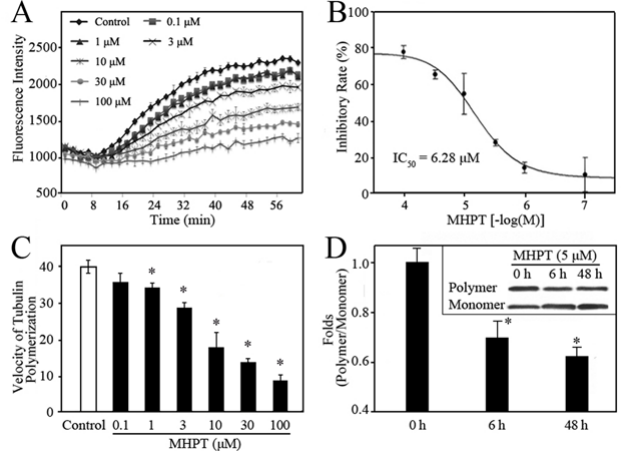
MHPT inhibited the polymerization of tubulin in a cell-free system (A-C) and in RD cells (D). (A) The tubulin polymerization process was monitored by fluorescence in the presence of MHPT at the indicated concentration. Microtubule formation after the addition of DMSO (control) generated a polymerization curve representing three phases: nucleation (0-8 min), growth (10–40 min), and equilibrium (40–60 min). (B) MHPT inhibited tubulin polymerization in a dose-dependent manner. The inhibitory rate was calculated as the ratio of the fluorescence change obtained with MHPT treatment to the fluorescence change of the control. The IC_50_ was the concentration at which 50% inhibition was achieved. (C) The velocity of tubulin polymerization in the growth phase. The polymerization velocity was calculated based on the ratio of the fluorescence change to the reaction time. *, *P* < 0.05 versus control. (D) Polymeric and monomeric tubulin fractions were isolated as described in the Methods. Equal amounts of total protein in each sample were analyzed by Western blot. The ratio of polymeric to monomeric tubulin was quantified by densitometric analysis using Image J software after normalization to the value at the 0 h time point. *, *P* < 0.05 versus 0 time point.

Microtubules are formed by the polymerization of two globular proteins, alpha- and beta-tubulin, which are essential for the maintenance of cell shape, cell proliferation and apoptosis [26–29]. Tubulin-targeting agents including paclitaxel and VCR can disrupt the polymerization of tubulin leading to cell cycle arrest followed by apoptosis [30–36]. The cytotoxicity of MHPT in RMS cells was caused by G2/M cell cycle arrest followed by apoptosis; this mechanism of cytotoxicity is the same as that observed for tubulin-targeting agents. To determine whether MHPT targets microtubules, the effect of MHPT on tubulin polymerization was monitored in a cell-free system. Alpha- and beta-tubulin subunits can polymerize and self-assemble to form microtubules in a time-dependent manner as shown by the control curve showed (Fig. 5A). MHPT moderately inhibited the formation of microtubules in a dose-dependent manner. The half maximal inhibitory concentration (IC_50_) of MHPT against tubulin polymerization was 6.28 μM (Fig. 5B). The velocity of microtubule formation in the growth phase (8–40 min) is calculated (Fig. 5C). It was found that as the treatment dose of MHPT increased, the velocity of microtubule formation decreased.

Tubulin exists in two forms in cells: polymeric (cytoskeletal) and monomeric (soluble). Tubulin polymerization inhibitors cause the accumulation of monomeric tubulin in cells. To confirm the inhibition of tubulin polymerization by MHPT, the polymeric and monomeric tubulin were extracted from RD cells and examined by Western blot. Monomeric tubulin expression was significantly higher than that of polymeric tubulin in RD cells after MHPT treatment compared with DMSO treatment from 6 to 48 h (Fig. 5D). This result indicated that MHPT can inhibit the assembly of microtubules. Furthermore, the time points at which MHPT treatment caused microtubule depolymerization and G2/M arrest in RD cells were consistent (from 6 to 48 h). Taken together, these results reveal that MHPT caused cytotoxicity in RMS cells by acting as a microtubule-depolymerizing agent.

### MHPT inhibits the growth of RD xenograft tumors *in vivo* without appreciable toxicity

Because MHPT showed impressive therapeutic efficacy *in vitro*, we further investigated its anti-tumor activity in an RD xenograft tumor model in nude mice. First, the acute toxicity of MHPT was evaluated in BALB/C mice to select a safe dose. Considering its limited solubility, a 200 mg/kg dose of MHPT (the highest available soluble concentration, intraperitoneal injection) was adopted. During the 2-week observation period, MHPT did not induce any noticeable toxicity, such as body weight loss, abnormal behavior, or changes in the organ/body weight ratio, blood biochemical data and hematological data (S3 Fig.).

**Fig 6:**
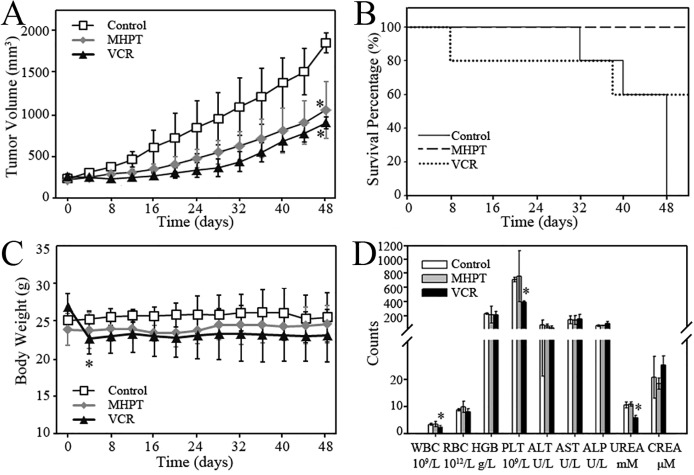
The anti-tumor efficacy of MHPT in an RD xenograft tumor model. (A) RD xenograft tumor growth curve in BALB/C nude mice. The mice were injected with the following: Vehicle (control, 5 doses, every other day), MHPT (40 mg/kg, 5 doses, every other day), or VCR (1 mg/kg, 5 doses, every 4 days). *, *P* < 0.05 versus the control group, n = 5. (B) Survival percentage of mice after administration. The mice were sacrificed when the tumors grew to nearly 2000 mm^3^ or other serious toxic signs were observed, including > 20% body weight loss. (C) The body weight of the mice was measured during the experiments. In the control and MHPT groups, the body weights of the mice were stable. In the VCR group, serious body weight loss occurred, and two mice died during experiments. *, *P* < 0.05 versus the body weights at day 0, n = 5. (D) Blood biochemical and hematological data for the BALB/C nude mice. There was no significant difference between the MHPT group and the control group. In VCR group, WBC, PLT and UREA were decreased significantly. *, *P* < 0.05 versus the control group, n = 5.

Next, the anti-tumor activity of MHPT *in vivo* was examined in an RD xenograft tumor model in BALB/C nude mice. Based on the dose used in the acute toxicity experiment, mice bearing RD xenograft tumors were intraperitoneally injected with five doses of 40 mg/kg MHPT. In solvent-treated group (control), RD xenograft tumors grew rapidly and achieved 1906 mm^3^ at day 48. Considering that the tumor volume may well exceed 2000 mm^3^ at the next time point, control group mice were sacrificed for animal welfare based on *in vivo* experiments criteria. Compared with the control group, MHPT greatly inhibited RD tumor growth by 48.6% (Fig. 6A). Additionally, MHPT did not cause appreciable toxicity (Figs. 6B-D). VCR and MHPT showed similar efficacy with respect to tumor inhibition (no statistic differences between VCR and MHPT group); however, VCR caused serious toxicity. Significant weight loss was observed in mice after VCR treatment (Fig. 6B), and two mice died during experiment (Fig. 6C): one died during drug administration, and the other was sacrificed at day 38 due to serious body weight loss. Moreover, white blood cells, platelets and blood urea were decreased significantly in mice treated with VCR (Fig. 6D). However, no pathological lesions were observed in major organs in each group (S4 Fig.). We suggest that the decreased blood urea after VCR treatment might be due to minimal intake of feed, which was the only protein source available to VCR treated mice suffering from drug toxicity. The other toxic effects of VCR observed in our study were consistent with those reported in literatures [24, 37, 38]. In summary, the growth of xenograft tumors after MHPT treatment was significantly inhibited, while tumors grew rapidly in the control group. All mice survived without appreciable toxicity after MHPT treatment, whereas lethal toxicity occurred after VCR treatment.

## Discussion

Microtubules are an attractive target for anticancer therapy. Microtubule-targeting agents suppress microtubule dynamics by stabilizing or depolymerizing microtubules, leading to disruption of the mitotic spindle in dividing cells, cell cycle arrest, and late apoptosis [30, 34]. Several microtubule targeting agents have been used clinically including paclitaxel (a microtubule stabilizing agent) and vinca alkaloids, vinblastine and VCR (microtubule-depolymerizing agents) [39]. These microtubule-targeting agents demonstrate excellent cytotoxicity; however, their dose-limiting toxicity has become a large roadblock to effective cancer therapy. They usually cause serious drug toxicity by blocking mitotic microtubule formation in normal cells, which greatly limits their clinical application. In this study, the clinical anti-RMS drug VCR showed very low selectivity *in vitro* and *in vivo*, as reported in the literatures [8–10, 24, 37]. In addition, it is difficult to synthesize these natural microtubule-targeting agents due to their complicated structures. Therefore, these agents tend to be very expensive, which limits their use, especially in developing countries.

In this study, MHPT was identified as a novel potent anti-RMS agent. MHPT significantly inhibited the growth of RMS cells *in vitro* and *in vivo*. The mechanistic study of the cytotoxicity of MHPT demonstrated that it functions as a microtubule- depolymerizing agent. MHPT exerts anti-RMS activity by suppressing the polymerization of tubulin, leading to RMS cell cycle arrest at the G2/M phase and, ultimately, apoptosis. This is consistent with the mode of action of other microtubule-depolymerizing agents [30–36]. In contrast to VCR, MHPT was much more highly selective for RMS cells. *In vitro*, the selectivity index of MHPT for RD and NHFB cells was higher than 227; this value was more than 12-fold higher than that of VCR. *In vivo*, MHPT suppressed 48.6% of the growth of RD tumors without appreciable toxicity, while VCR caused lethal toxicity including serious weight loss, blood toxicity, or even death when similar tumor growth inhibition was achieved.

Both MHPT and VCR target tubulin; however, MHPT showed much lower toxicity *in vitro* and *in vivo* under our experimental conditions. The major mechanism of drug toxicity is an exaggerated pharmacological impact on the target [40]. Microtubules in cancer cells which proliferate rapidly are much more active and dynamic than microtubules in normal cells. The differences in selectivity between MHPT and VCR might be due to the fact that MHPT has a more moderate inhibitory effect against tubulin (IC_50_ = 6.28 μM) compared with VCR (IC_50_ = 0.43 μM) [41]. We speculate that as a moderate tubulin polymerization inhibitor, MHPT requires a more dynamic tubulin turn-over to inhibit the cell cycle, and this scenario only exists in cancer cells. Therefore, the cell cycle inhibitory effect of MHPT may be adequate to cause cytotoxicity in cancer cells but not robust enough to induce similar cytotoxicity in normal cells. This tentative explanation is also in line with several recent reports [13, 42, 43].

In addition, off-target effects of drugs, which are caused by the interaction between drugs and unintended targets or signaling pathways, can also cause drug toxicity [40, 44–50]. Almost all signaling pathways are mediated by protein kinases, which constitute the most functional and largest protein family [51]. The unintended inhibition of protein kinases by drugs leads to serious drug toxicity as reported previously [44–47]. Thus, we assessed the ability of MHPT to inhibit a panel of 433 human kinases (covering 80% of the human protein kinome) using the KINOMEscan platform [52]. MHPT did not noticeably inhibit any of 433 kinases screened (S1 Table), which indicates that MHPT does not disturb the normal cellular functions mediated by kinases.

In summary, MHPT exhibited potent anti-RMS activity *in vitro* and *in vivo* without appreciable toxicity in this study. These results suggested that MHPT may be further developed as a low-toxicity anticancer agent for RMS chemotherapy.

## Supporting Information

S1 Table Screen to determine the effects of MHPT on kinases. (DOCX)

S1 Fig MHPT caused time-dependent cell cycle arrest in RD cells. (DOCX)

S2 Fig MHPT induced RD cell apoptosis in a time-dependent manner. (DOCX)

S3 Fig The acute toxicity of MHPT in BALB/C mice. (DOCX)

S4 Fig Histopathologic examination of the major organs of the mice bearing RD xenograft tumors at the end of the experiment. (DOCX)
