# Supplementary material for: The Novel Tubulin Polymerization Inhibitor MHPT Exhibits Selective Anti-Tumor Activity against Rhabdomyosarcoma In Vitro and In Vivo
Source: PLoS One. 2015 Mar 26;10(3):e0121806. doi: 10.1371/journal.pone.0121806 (PMC4374867; doi:10.1371/journal.pone.0121806)
Supplement: S1 Table — (DOCX) [file pone.0121806.s001.docx]

**S1 Table. Screen to determine the effects of MHPT on kinases**

| **Kinase Target** | **% Ctrl­*** | **Kinase Target** | **% Ctrl** |
| --- | --- | --- | --- |
| AAK1 | 93 | MAP3K15 | 100 |
| ABL1(E255K)-phosphorylated | 100 | MAP3K2 | 100 |
| ABL1(F317I)-nonphosphorylated | 100 | MAP3K3 | 57 |
| ABL1(F317I)-phosphorylated | 44 | MAP3K4 | 99 |
| ABL1(F317L)-nonphosphorylated | 100 | MAP4K2 | 75 |
| ABL1(F317L)-phosphorylated | 51 | MAP4K3 | 62 |
| ABL1(H396P)-nonphosphorylated | 52 | MAP4K4 | 82 |
| ABL1(H396P)-phosphorylated | 90 | MAP4K5 | 100 |
| ABL1(M351T)-phosphorylated | 72 | MAPKAPK2 | 100 |
| ABL1(Q252H)-nonphosphorylated | 100 | MAPKAPK5 | 97 |
| ABL1(Q252H)-phosphorylated | 67 | MARK1 | 100 |
| ABL1(T315I)-nonphosphorylated | 100 | MARK2 | 80 |
| ABL1(T315I)-phosphorylated | 97 | MARK3 | 100 |
| ABL1(Y253F)-phosphorylated | 70 | MARK4 | 100 |
| ABL1-nonphosphorylated | 100 | MAST1 | 100 |
| ABL1-phosphorylated | 66 | MEK1 | 97 |
| ABL2 | 98 | MEK2 | 100 |
| ACVR1 | 100 | MEK3 | 75 |
| ACVR1B | 88 | MEK4 | 100 |
| ACVR2A | 85 | MEK6 | 100 |
| ACVR2B | 67 | MELK | 100 |
| ACVRL1 | 100 | MERTK | 95 |
| ADCK3 | 98 | MET | 100 |
| ADCK4 | 64 | MET(M1250T) | 100 |
| AKT1 | 100 | MET(Y1235D) | 100 |
| AKT2 | 93 | MINK | 72 |
| AKT3 | 100 | MKK7 | 77 |
| ALK | 100 | MKNK1 | 100 |
| AMPK-alpha1 | 92 | MKNK2 | 80 |
| AMPK-alpha2 | 69 | MLCK | 63 |
| ANKK1 | 94 | MLK1 | 73 |
| ARK5 | 100 | MLK2 | 100 |
| ASK1 | 100 | MLK3 | 99 |
| ASK2 | 100 | MRCKA | 100 |
| AURKA | 90 | MRCKB | 100 |
| AURKB | 88 | MST1 | 100 |
| AURKC | 100 | MST1R | 100 |
| AXL | 91 | MST2 | 53 |
| BIKE | 100 | MST3 | 89 |
| BLK | 28 | MST4 | 100 |
| BMPR1A | 89 | MTOR | 98 |
| BMPR1B | 66 | MUSK | 88 |
| BMPR2 | 63 | MYLK | 51 |
| BMX | 100 | MYLK2 | 86 |
| BRAF | 92 | MYLK4 | 66 |
| BRAF(V600E) | 74 | MYO3A | 91 |
| BRK | 76 | MYO3B | 75 |
| BRSK1 | 87 | NDR1 | 99 |
| BRSK2 | 97 | NDR2 | 100 |
| BTK | 100 | NEK1 | 100 |
| CAMK1 | 91 | NEK11 | 91 |
| CAMK1D | 95 | NEK2 | 100 |
| CAMK1G | 85 | NEK3 | 80 |
| CAMK2A | 80 | NEK4 | 85 |
| CAMK2B | 92 | NEK5 | 70 |
| CAMK2D | 76 | NEK6 | 95 |
| CAMK2G | 100 | NEK7 | 100 |
| CAMK4 | 86 | NEK9 | 100 |
| CAMKK1 | 91 | NIM1 | 100 |
| CAMKK2 | 100 | NLK | 90 |
| CASK | 100 | OSR1 | 100 |
| CDC2L1 | 100 | p38-alpha | 84 |
| CDC2L2 | 100 | p38-beta | 100 |
| CDC2L5 | 85 | p38-delta | 75 |
| CDK11 | 77 | p38-gamma | 100 |
| CDK2 | 91 | PAK1 | 100 |
| CDK3 | 87 | PAK2 | 85 |
| CDK4-cyclinD1 | 73 | PAK3 | 100 |
| CDK4-cyclinD3 | 89 | PAK4 | 100 |
| CDK5 | 100 | PAK6 | 94 |
| CDK7 | 62 | PAK7 | 85 |
| CDK8 | 100 | PCTK1 | 74 |
| CDK9 | 76 | PCTK2 | 86 |
| CDKL1 | 100 | PCTK3 | 100 |
| CDKL2 | 90 | PDPK1 | 100 |
| CDKL3 | 100 | PFCDPK1(P.falciparum) | 100 |
| CDKL5 | 73 | PFPK5(P.falciparum) | 100 |
| CHEK1 | 87 | PFTAIRE2 | 79 |
| CHEK2 | 100 | PFTK1 | 100 |
| CLK1 | 45 | PHKG1 | 100 |
| CLK2 | 46 | PHKG2 | 94 |
| CLK3 | 100 | PIK3C2B | 94 |
| CLK4 | 25 | PIK3C2G | 79 |
| CSF1R | 68 | PIK3CA | 100 |
| CSK | 100 | PIK3CA(C420R) | 100 |
| CSNK1A1 | 55 | PIK3CA(E542K) | 93 |
| CSNK1A1L | 75 | PIK3CA(E545A) | 100 |
| CSNK1G1 | 100 | PIK3CA(E545K) | 100 |
| CSNK1G2 | 76 | PIK3CA(H1047L) | 100 |
| CSNK1G3 | 100 | PIK3CA(H1047Y) | 100 |
| CTK | 57 | PIK3CA(I800L) | 87 |
| DAPK1 | 55 | PIK3CA(M1043I) | 100 |
| DAPK2 | 52 | PIK3CA(Q546K) | 80 |
| DAPK3 | 38 | PIK3CB | 100 |
| DCAMKL1 | 98 | PIK3CD | 73 |
| DCAMKL2 | 100 | PIK3CG | 92 |
| DCAMKL3 | 86 | PIK4CB | 79 |
| DDR1 | 75 | PIM1 | 35 |
| DDR2 | 78 | PIM2 | 11 |
| DLK | 82 | PIM3 | 36 |
| DMPK | 65 | PIP5K1A | 97 |
| DMPK2 | 97 | PIP5K1C | 42 |
| DRAK1 | 24 | PIP5K2B | 55 |
| DRAK2 | 69 | PIP5K2C | 67 |
| DYRK1A | 32 | PKAC-alpha | 100 |
| DYRK1B | 94 | PKAC-beta | 94 |
| DYRK2 | 64 | PKMYT1 | 82 |
| EGFR | 58 | PKN1 | 43 |
| EGFR(E746-A750del) | 26 | PKN2 | 71 |
| EGFR(G719C) | 27 | PKNB(M.tuberculosis) | 100 |
| EGFR(G719S) | 55 | PLK1 | 100 |
| EGFR(L747-E749del, A750P) | 39 | PLK2 | 100 |
| EGFR(L747-S752del, P753S) | 36 | PLK3 | 100 |
| EGFR(L747-T751del,Sins) | 80 | PLK4 | 98 |
| EGFR(L858R) | 40 | PRKCD | 74 |
| EGFR(L858R,T790M) | 98 | PRKCE | 82 |
| EGFR(L861Q) | 73 | PRKCH | 51 |
| EGFR(S752-I759del) | 100 | PRKCI | 78 |
| EGFR(T790M) | 93 | PRKCQ | 100 |
| EIF2AK1 | 88 | PRKD1 | 95 |
| EPHA1 | 96 | PRKD2 | 95 |
| EPHA2 | 97 | PRKD3 | 100 |
| EPHA3 | 73 | PRKG1 | 96 |
| EPHA4 | 100 | PRKG2 | 88 |
| EPHA5 | 100 | PRKR | 88 |
| EPHA6 | 100 | PRKX | 100 |
| EPHA7 | 100 | PRP4 | 82 |
| EPHA8 | 100 | PYK2 | 91 |
| EPHB1 | 86 | QSK | 99 |
| EPHB2 | 100 | RAF1 | 88 |
| EPHB3 | 100 | RET | 93 |
| EPHB4 | 100 | RET(M918T) | 100 |
| EPHB6 | 44 | RET(V804L) | 97 |
| ERBB2 | 69 | RET(V804M) | 58 |
| ERBB3 | 92 | RIOK1 | 75 |
| ERBB4 | 48 | RIOK2 | 92 |
| ERK1 | 81 | RIOK3 | 87 |
| ERK2 | 100 | RIPK1 | 96 |
| ERK3 | 97 | RIPK2 | 45 |
| ERK4 | 100 | RIPK4 | 100 |
| ERK5 | 100 | RIPK5 | 67 |
| ERK8 | 97 | ROCK1 | 64 |
| ERN1 | 85 | ROCK2 | 45 |
| FAK | 93 | ROS1 | 94 |
| FER | 100 | RPS6KA4(Kin.Dom.1-N-terminal) | 88 |
| FES | 100 | RPS6KA4(Kin.Dom.2-C-terminal) | 52 |
| FGFR1 | 93 | RPS6KA5(Kin.Dom.1-N-terminal) | 83 |
| FGFR2 | 98 | RPS6KA5(Kin.Dom.2-C-terminal) | 87 |
| FGFR3 | 92 | RSK1(Kin.Dom.1-N-terminal) | 63 |
| FGFR3(G697C) | 95 | RSK1(Kin.Dom.2-C-terminal) | 58 |
| FGFR4 | 82 | RSK2(Kin.Dom.1-N-terminal) | 66 |
| FGR | 76 | RSK3(Kin.Dom.1-N-terminal) | 97 |
| FLT1 | 86 | RSK3(Kin.Dom.2-C-terminal) | 80 |
| FLT3 | 23 | RSK4(Kin.Dom.1-N-terminal) | 100 |
| FLT3(D835H) | 47 | RSK4(Kin.Dom.2-C-terminal) | 71 |
| FLT3(D835Y) | 22 | S6K1 | 85 |
| FLT3(ITD) | 70 | SBK1 | 89 |
| FLT3(K663Q) | 32 | SgK110 | 100 |
| FLT3(N841I) | 57 | SGK3 | 100 |
| FLT3(R834Q) | 96 | SIK | 100 |
| FLT4 | 100 | SIK2 | 74 |
| FRK | 88 | SLK | 100 |
| FYN | 94 | SNARK | 86 |
| GAK | 39 | SNRK | 100 |
| GCN2(Kin.Dom.2,S808G) | 100 | SRC | 44 |
| GRK1 | 100 | SRMS | 100 |
| GRK4 | 100 | SRPK1 | 100 |
| GRK7 | 32 | SRPK2 | 100 |
| GSK3A | 87 | SRPK3 | 94 |
| GSK3B | 96 | STK16 | 100 |
| HCK | 16 | STK33 | 89 |
| HIPK1 | 42 | STK35 | 95 |
| HIPK2 | 22 | STK36 | 48 |
| HIPK3 | 36 | STK39 | 79 |
| HIPK4 | 89 | SYK | 74 |
| HPK1 | 70 | TAK1 | 49 |
| HUNK | 60 | TAOK1 | 91 |
| ICK | 100 | TAOK2 | 73 |
| IGF1R | 96 | TAOK3 | 81 |
| IKK-alpha | 93 | TBK1 | 100 |
| IKK-beta | 100 | TEC | 100 |
| IKK-epsilon | 82 | TESK1 | 95 |
| INSR | 94 | TGFBR1 | 66 |
| INSRR | 69 | TGFBR2 | 100 |
| IRAK1 | 85 | TIE1 | 100 |
| IRAK3 | 51 | TIE2 | 94 |
| IRAK4 | 100 | TLK1 | 97 |
| ITK | 100 | TLK2 | 91 |
| JAK1(JH1domain-catalytic) | 100 | TNIK | 61 |
| JAK1(JH2domain-pseudokinase) | 88 | TNK1 | 100 |
| JAK2(JH1domain-catalytic) | 82 | TNK2 | 100 |
| JAK3(JH1domain-catalytic) | 54 | TNNI3K | 90 |
| JNK1 | 94 | TRKA | 79 |
| JNK2 | 81 | TRKB | 73 |
| JNK3 | 69 | TRKC | 69 |
| KIT(A829P) | 69 | TRPM6 | 100 |
| KIT(D816H) | 85 | TSSK1B | 93 |
| KIT(D816V) | 38 | TTK | 90 |
| KIT(L576P) | 27 | TXK | 83 |
| KIT(V559D) | 12 | TYK2(JH1domain-catalytic) | 74 |
| KIT(V559D,T670I) | 89 | TYK2(JH2domain-pseudokinase) | 75 |
| KIT(V559D,V654A) | 36 | TYRO3 | 82 |
| LATS1 | 89 | ULK1 | 75 |
| LATS2 | 75 | ULK2 | 100 |
| LCK | 43 | ULK3 | 98 |
| LIMK1 | 100 | VEGFR2 | 75 |
| LIMK2 | 96 | VRK2 | 100 |
| LKB1 | 67 | WEE1 | 100 |
| LOK | 100 | WEE2 | 100 |
| LRRK2 | 100 | YANK1 | 100 |
| LRRK2(G2019S) | 95 | YANK2 | 73 |
| LTK | 100 | YANK3 | 100 |
| LYN | 65 | YES | 70 |
| LZK | 100 | YSK1 | 100 |
| MAK | 82 | YSK4 | 96 |
| MAP3K1 | 82 | ZAK | 98 |
|  |  | ZAP70 | 89 |

*In this assay, MHPT was screened at a concentration of 10 μM. The selectivity score (% Ctrl < 10) of MHPT was zero. MHPT did not noticeably inhibit any of the 433 kinases screened.

**Methods description**

**Kinase screening assay.** MHPT was prepared in DMSO and sent to DiscoverRx Corporation for the KINOMEscan’s assay. KINOMEscan is based on a competition assay that quantitatively measure the ability of a test compound to compete with an active site-directed ligand for binding to the kinase active site using the KINOMEscan platform. Binding reactions were assembled at room temperature using DNA-tagged kinases, immobilized affinity ligand and MHPT in binding buffer (20% SeaBlock, 0.17× PBS, 0.05% Tween 20, and 6 mM DTT). The assay plates were incubated with shaking for 1 h, and the liganded beads were washed with wash buffer (1× PBS, 0.05% Tween 20). The beads were then re-suspended in elution buffer (1× PBS, 0.05% Tween 20, 0.5 μM ligand) and incubated with shaking for 30 min. The kinase concentration in the eluates was measured by qPCR. The results are represented as “% Ctrl”, which was calculated using the following formula: (test compound signal− positive control signal)/(DMSO signal–positive control signal)×100. A lower % Ctrl indicates stronger binding to the kinase active site. Kinases with % Ctrl <10 were selected as kinases that were inhibited strongly by the test compound.
