# Supplementary material for: The Novel Tubulin Polymerization Inhibitor MHPT Exhibits Selective Anti-Tumor Activity against Rhabdomyosarcoma In Vitro and In Vivo
Source: PLoS One. 2015 Mar 26;10(3):e0121806. doi: 10.1371/journal.pone.0121806 (PMC4374867; doi:10.1371/journal.pone.0121806)
Supplement: S1 Fig — (DOCX) [file pone.0121806.s002.docx]

**
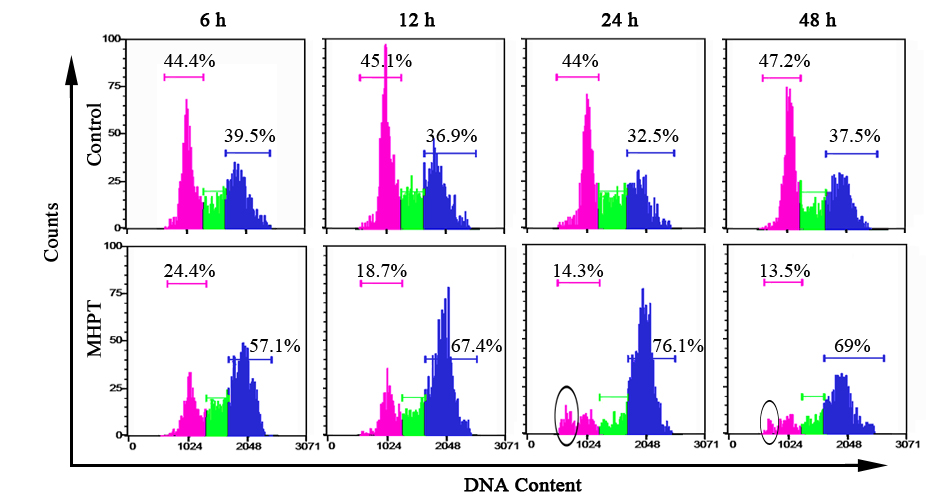
 S1 Fig.** **MHPT caused time-dependent cell cycle arrest in RD cells.** Cells were treated with DMSO (control) or MHPT (5 μM) for 6, 12, 24, and 48 h. The pink areas indicate arrest at the G1/G0 phase; the green areas indicate arrest at S phase; the blue areas indicate arrest at G2/M phase; and the circled areas indicate arrest at sub-G1 phase.
