# Supplementary material for: The Novel Tubulin Polymerization Inhibitor MHPT Exhibits Selective Anti-Tumor Activity against Rhabdomyosarcoma In Vitro and In Vivo
Source: PLoS One. 2015 Mar 26;10(3):e0121806. doi: 10.1371/journal.pone.0121806 (PMC4374867; doi:10.1371/journal.pone.0121806)
Supplement: S2 Fig — (DOCX) [file pone.0121806.s003.docx]

**
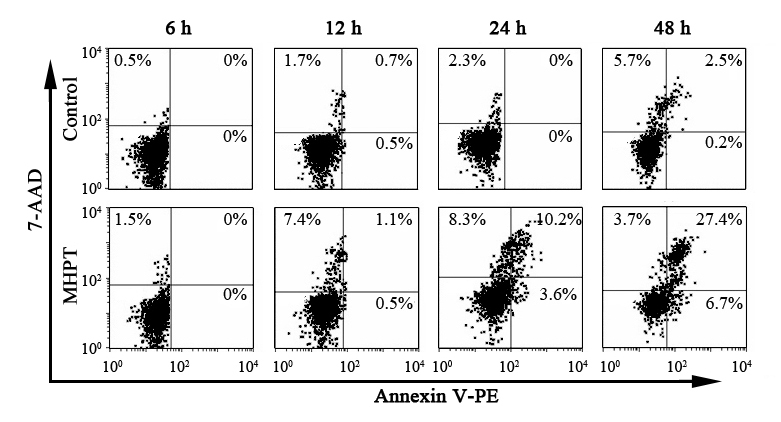
**

**S2 Fig. MHPT induced RD cell apoptosis in a time-dependent manner.** RD cells were treated with DMSO (control) and MHPT (5 μM) for 6, 12, 24, and 48 h. RD cells were stained with Guava Nexin regent containing 7-AAD/Annexin-V-PE and analyzed by flow cytometry. Among the four windows of each plot, the lower left indicates normal cells, the lower right indicates early apoptotic cells, and the upper right indicates late phase apoptotic cells or necrotic cells.
