# Supplementary material for: The Novel Tubulin Polymerization Inhibitor MHPT Exhibits Selective Anti-Tumor Activity against Rhabdomyosarcoma In Vitro and In Vivo
Source: PLoS One. 2015 Mar 26;10(3):e0121806. doi: 10.1371/journal.pone.0121806 (PMC4374867; doi:10.1371/journal.pone.0121806)
Supplement: S3 Fig — (DOCX) [file pone.0121806.s004.docx]

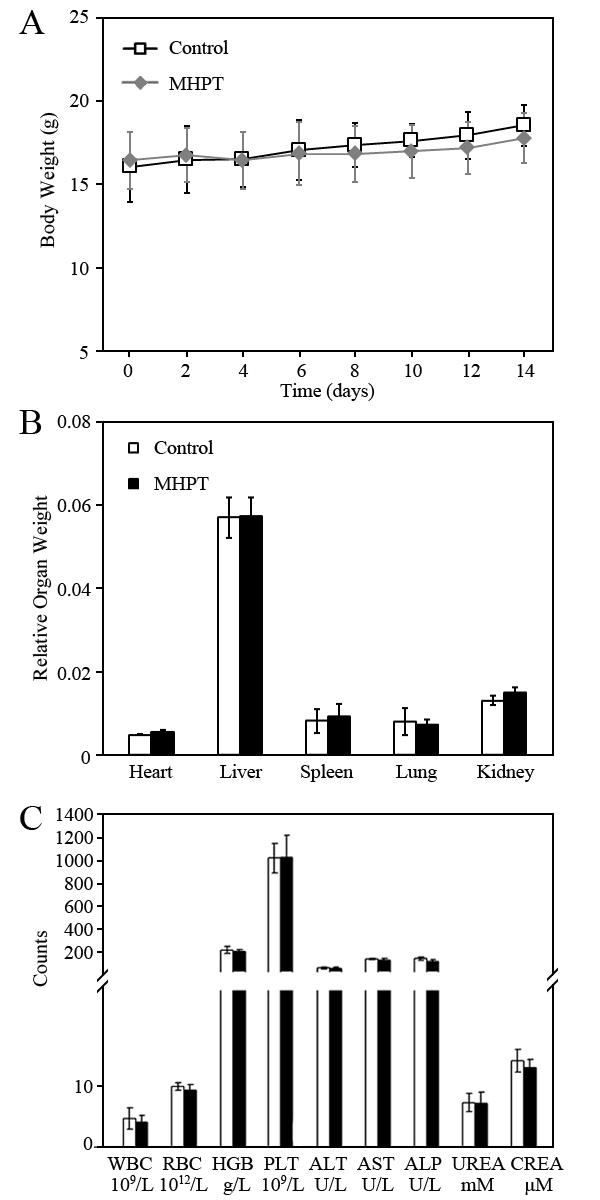


**S3 Fig. The acute toxicity of MHPT in BALB/C mice.** A single 200 mg/kg dose of MHPT was injected intraperitoneally on day 0. (A) The body weight of mice was measured during the experiment. (B) Relative organ weight (the ratio of organ weight to body weight) was calculated to evaluate organ abnormalities at the end of the experiment. (C) Blood biochemical and hematological analyses of the mice were performed at the end of experiment. There were no significant differences between the MHPT and the control groups in A-C by Student’s *t-*test (n=4, *p*< 0.05).

**Methods description**

**Acute toxicity testing.** BALB/C mice (female, 4 weeks old) were purchased from Vital River Research Animal Services (Beijing, China). After one week of acclimatization, the mice were randomized into two groups: Control (vehicle including 85% PBS, and 15% PPG) and MHPT. Considering the limited solubility, the mice were administered at a single dose of 200 mg/kg MHPT or the same volume of vehicle by intraperitoneal injection. The mice were observed for 14 days during which mortality, body weight loss, and other abnormal clinical signs were monitored. At day 14, the mice were euthanized and sacrificed. The blood of each mouse was collected and analyzed for blood biochemical and hematological analyses. Necropsies were performed and organ/body weight coefficients were measured.
