# Supplementary material for: The Novel Tubulin Polymerization Inhibitor MHPT Exhibits Selective Anti-Tumor Activity against Rhabdomyosarcoma In Vitro and In Vivo
Source: PLoS One. 2015 Mar 26;10(3):e0121806. doi: 10.1371/journal.pone.0121806 (PMC4374867; doi:10.1371/journal.pone.0121806)
Supplement: S4 Fig — (DOCX) [file pone.0121806.s005.docx]

**S4**
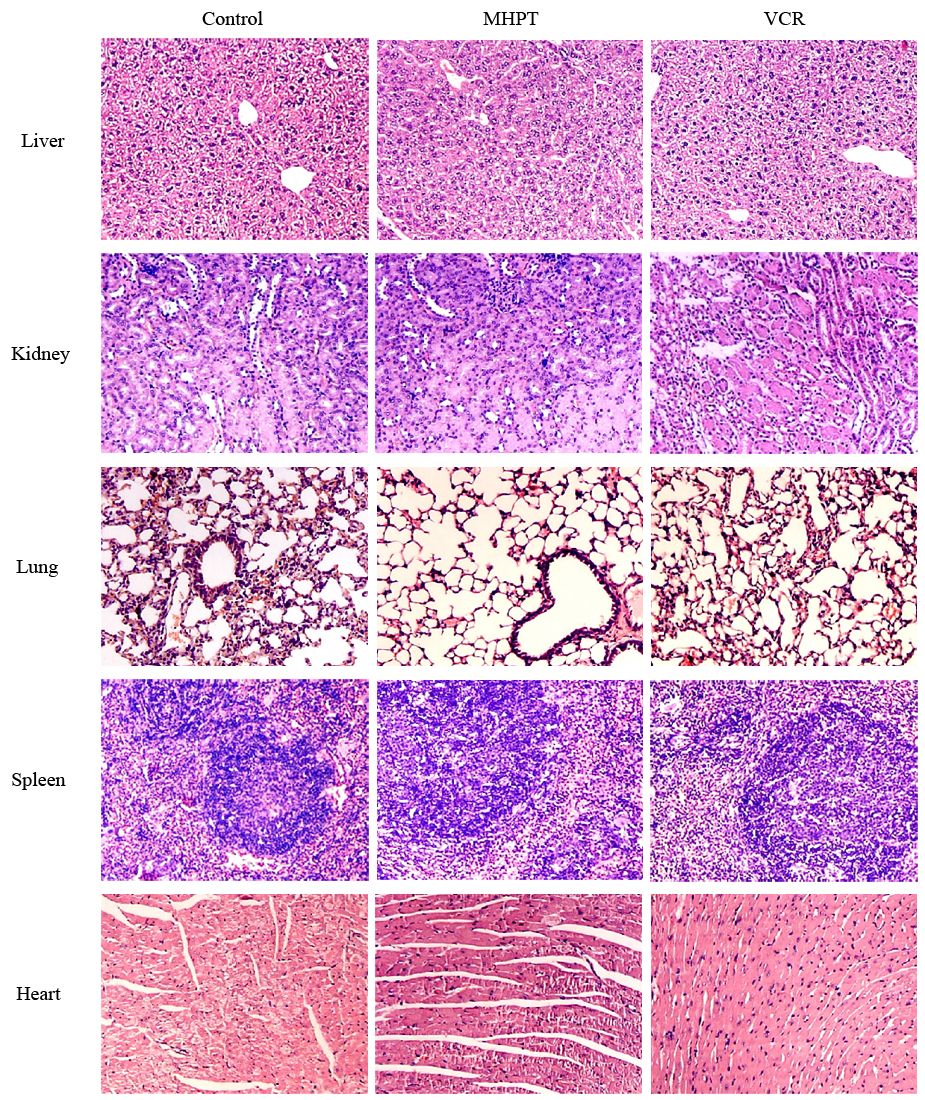
 **Fig. Histopathologic examination of the major organs of the mice bearing RD xenograft tumors at the end of the experiment.** No pathological changes were observed in any of the groups. Magnification 200×.

**Methods description**

**Acute toxicity testing.** BALB/C mice (female, 4 weeks old) were purchased from Vital River Research Animal Services (Beijing, China). After one week of acclimatization, the mice were randomized into two groups: Control (vehicle including 85% PBS, and 15% PPG) and MHPT. Considering the limited solubility, the mice were administered at a single dose of 200 mg/kg MHPT or the same volume of vehicle by intraperitoneal injection. The mice were observed for 14 days during which mortality, body weight loss, and other abnormal clinical signs were monitored. At day 14, the mice were euthanized and sacrificed. The blood of each mouse was collected and analyzed for blood biochemical and hematological analyses. Necropsies were performed and organ/body weight coefficients were measured.
